# 1162. Influenza Vaccination Among Pregnant People in the Vaccine Safety Datalink, 2016-17 through 2022-23 Influenza Seasons

**DOI:** 10.1093/ofid/ofad500.1002

**Published:** 2023-11-27

**Authors:** Stephanie Irving, Bradley Crane, Eric Weintraub, Tia L Kauffman, Neon Brooks, Suchita A Patel, Hilda Razzaghi, Edward Belongia, Matthew F Daley, Darios Getahun, Sungching C Glenn, Simon Hambidge, Lisa Jackson, Elyse Kharbanda, Nicola P Klein, Ousseny Zerbo, Allison L Naleway

**Affiliations:** Kaiser Permanente Center for Health Research, Portland, Oregon; Kaiser Permanente Center for Health Research, Portland, Oregon; Centers for Disease Control and Prevention, Atlanta, GA; Kaiser Permanente Center for Health Research, Portland, Oregon; Kaiser Permanente Center for Health Research, Portland, Oregon; Centers for Disease Control and Prevention, Atlanta, GA; CDC, Atlanta, Georgia; Marshfield Clinic Research Institute, Marshfield, WI; Kaiser Permanente Colorado, Aurora, Colorado; Kaiser Permanente Southern California, Pasadena, California; Kaiser Permanente Southern California, Pasadena, California; Denver Health, Denver, CO; Kaiser Permanente Washington Health Research Institute, Seattle, WA; HealthPartners Institute, Minneapolis, Minnesota; Kaiser Permanente Northern California, Oakland, California; Division of Research Kaiser Permanente Vaccine Study Center, Oakland, California; Kaiser Permanente Center for Health Research, Portland, Oregon

## Abstract

**Background:**

Estimates of influenza vaccination coverage during pregnancy vary, but studies in the US prior to the 2020-21 influenza season show increasing coverage over time. However, there are limited data on influenza vaccination coverage among pregnant people during the COVID-19 pandemic.

**Methods:**

Within the Vaccine Safety Datalink, we examined influenza vaccination coverage among people ages 18-49 years identified as pregnant with a live birth using a validated algorithm. For primary analyses covering the 2016-17 through 2021-22 influenza seasons, we captured all influenza vaccination between July 1 and March 31 of each season, irrespective of the timing of administration relative to pregnancy (i.e., prior to, during, or after pregnancy), and assessed crude coverage; demographic and clinical characteristics associated with vaccination; and vaccination patterns by calendar month of pregnancy start. Secondary analyses included crude coverage estimates for the 2022-23 season, using data through January 2023, stratified by race and ethnicity.

**Results:**

In primary analyses, among cohorts of pregnant people ranging from 128,267 to 139,927 each season, crude annual influenza vaccination coverage ranged from a high of 71% (2019-20) to a low of 56% (2021-22). In each of the six seasons, coverage was lowest among 18-24-year-olds (Figure) and among non-Hispanic Black pregnant people. The 2021-22 season had the lowest coverage estimates across all age and race and ethnicity groups. Coverage differed based on the calendar month of pregnancy onset, with the lowest coverage observed among pregnancies starting in January-March during each influenza season. In secondary analyses, overall coverage as of January 2023 was 47.9%, a decrease of 7.7 percentage points from January 2022 estimates.

**Figure:**
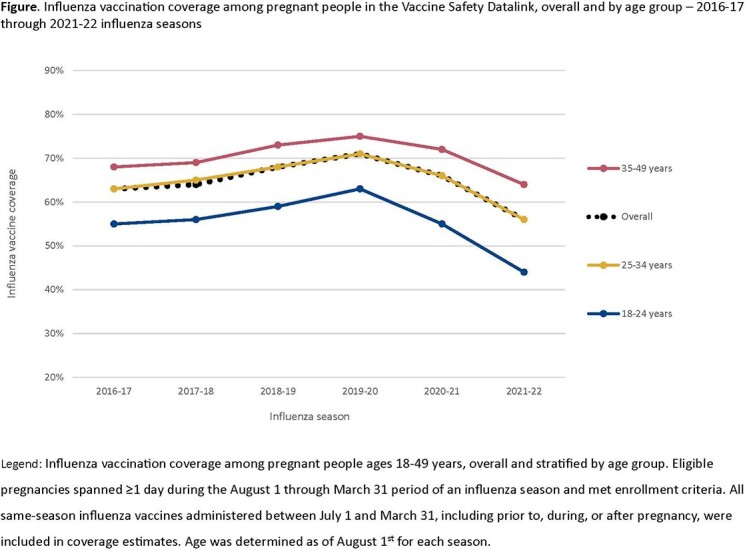

**Conclusion:**

Influenza vaccination coverage among all pregnant people increased from the 2016-17 influenza season through the 2019-20 influenza season, then decreased to the lowest level in the 2021-22 season. Interim estimates for the 2022-23 season declined further. The decreases seen in recent seasons, likely due in part to the COVID-19 pandemic, were consistent across all characteristics examined, and highlight the need for continued efforts to improve influenza vaccination coverage in pregnant people.

**Disclosures:**

**Edward Belongia, MD**, Seqirus: Grant/Research Support **Lisa Jackson, MD, MPH**, Pfizer: Grant/Research Support **Nicola P. Klein, MD, PhD**, GlaxoSmithKline: Grant/Research Support|Merck: Grant/Research Support|Pfizer: Grant/Research Support|Sanofi Pasteur: Grant/Research Support

